# Identification of copper metabolism-related subtypes and establishment of the prognostic model in ovarian cancer

**DOI:** 10.3389/fendo.2023.1145797

**Published:** 2023-03-06

**Authors:** Songyun Zhao, Xin Zhang, Feng Gao, Hao Chi, Jinhao Zhang, Zhijia Xia, Chao Cheng, Jinhui Liu

**Affiliations:** ^1^ Wuxi Medical Center of Nanjing Medical University, Wuxi, China; ^2^ Department of Neurosurgery, Wuxi People's Hospital Affiliated to Nanjing Medical University, Wuxi, China; ^3^ Department of Pathology, The Second People's Hospital of Foshan, Affiliated Foshan Hospital of Southern Medical University, Foshan, China; ^4^ Department of Orthopaedics, The First Affiliated Hospital of Nanjing Medical University, Nanjing, China; ^5^ Southwest Medical University, Luzhou, China; ^6^ Department of General, Visceral, and Transplant Surgery, Ludwig-Maximilians University, Munich, Germany; ^7^ Department of Gynecology, The First Affiliated Hospital of Nanjing Medical University, Nanjing, China

**Keywords:** copper metabolism, OC, machine learning, Tumor microenvironment, immunotherapy, risk score signature

## Abstract

**Background:**

Ovarian cancer (OC) is one of the most common and most malignant gynecological malignancies in gynecology. On the other hand, dysregulation of copper metabolism (CM) is closely associated with tumourigenesis and progression. Here, we investigated the impact of genes associated with copper metabolism (CMRGs) on the prognosis of OC, discovered various CM clusters, and built a risk model to evaluate patient prognosis, immunological features, and therapy response.

**Methods:**

15 CMRGs affecting the prognosis of OC patients were identified in The Cancer Genome Atlas (TCGA). Consensus Clustering was used to identify two CM clusters. lasso-cox methods were used to establish the copper metabolism-related gene prognostic signature (CMRGPS) based on differentially expressed genes in the two clusters. The GSE63885 cohort was used as an external validation cohort. Expression of CM risk score-associated genes was verified by single-cell sequencing and quantitative real-time PCR (qRT-PCR). Nomograms were used to visually depict the clinical value of CMRGPS. Differences in clinical traits, immune cell infiltration, and tumor mutational load (TMB) between risk groups were also extensively examined. Tumour Immune Dysfunction and Rejection (TIDE) and Immune Phenotype Score (IPS) were used to validate whether CMRGPS could predict response to immunotherapy in OC patients.

**Results:**

In the TCGA and GSE63885 cohorts, we identified two CM clusters that differed significantly in terms of overall survival (OS) and tumor microenvironment. We then created a CMRGPS containing 11 genes to predict overall survival and confirmed its reliable predictive power for OC patients. The expression of CM risk score-related genes was validated by qRT-PCR. Patients with OC were divided into low-risk (LR) and high-risk (HR) groups based on the median CM risk score, with better survival in the LR group. The 5-year AUC value reached 0.74. Enrichment analysis showed that the LR group was associated with tumor immune-related pathways. The results of TIDE and IPS showed a better response to immunotherapy in the LR group.

**Conclusion:**

Our study, therefore, provides a valuable tool to further guide clinical management and tailor the treatment of patients with OC, offering new insights into individualized treatment.

## Introduction

Ovarian cancer (OC) continues to be the primary cause of cancer mortality among the most prevalent gynecological malignancies globally. Because of its extremely high mortality rate, it has become a major threat to women’s reproductive health (1). Ovarian cancer is often diagnosed at a late stage because patients are often asymptomatic in the early stages, losing the best opportunity for treatment (2, 3). The standard treatment for ovarian cancer is surgical resection supplemented by chemotherapy with cisplatin (4). In recent years, although some progress has been made in chemotherapy and biological therapy for ovarian cancer, the five-year survival rate for patients is still around 30% (3).

Copper (Cu) is an indispensable micronutrient for the development and replication of all eukaryotes (5). As a transition element, the valence transition of Cu affects to some extent the redox state of cells and is closely related to oxidative stress, mitochondrial function, and programmed cell death (6). Thus, the link between copper and tumors has attracted the interest of researchers, with tumors requiring higher levels of copper compared to healthy tissue (7, 8). Elevated copper concentrations in tumors or serum have been reported in patients with a variety of cancers, including breast, lung, thyroid, gynecological, and prostate cancers (9–12). Copper metabolism imbalances modify lipid, glycolysis, and insulin resistance in addition to the mitochondrial respiration process (13). Copper can also promote tumor angiogenesis leading to tumor development, growth, and metastasis (14). Recently, it has been demonstrated that copper can control the expression of the immune-evading protein programmed death ligand 1 (PD-L1) on the surface of certain cancer cells (15).

A growing number of observations link imbalances in copper metabolism to tumor growth and metastasis in cancer. Also, more results suggest that copper imbalance leads to a decreased immune response to tumor cells. However, there is a need to establish more biomarkers related to copper metabolism and to further link copper-dependent targets and pathways to tumor susceptibility. A bioinformatics-based analysis has identified CMRGs as potential prognostic biomarkers for lung cancer (16). Therefore, identifying different clustering profiles and establishing CM-related signatures may be an effective means to predict prognosis and immunotherapeutic response in patients with ovarian cancer.

477 ovarian cancer samples and 133 CMRGs were acquired for this investigation from the TCGA, GEO, and MSigDB databases, respectively. We identified 15 CMRGs with prognostic significance for OC, examined the gene expression profiles and mutational patterns of the 15 CMRGs in OC, and divided the ovarian cancer population into two distinct CM clusters. Following the development of a predictive model based on the differentially expressed genes (DEGs) between the CM clusters, patients in the LR group had a better prognosis and were more likely to have better immunotherapy results.

## Materials and methods

### Data sources

Gene expression profiles (fragments per kilobase million (FPKM) and related clinicopathological data for OC were downloaded from the Gene Expression Omnibus (GEO) and The Cancer Genome Atlas (TCGA) databases. The GEO cohort (GSE63885, GSE9891) and TCGA-OV cohort were obtained for subsequent analysis. Fpkm was transformed into transcripts per kilobase million (TPM) and TPM was considered identical to transcripts from the GEO microarray (17). The “sva” algorithm combined the two datasets, eliminating the batch effect. 376 cases from the TCGA cohort and 101 OC patients from the GEO cohort were included in the follow-up analysis.

Copper metabolism-related genes (**Supplementary Table 1**) were downloaded from the MsigDB (18). The merged TCGA-GTEx cohort was downloaded from the UCSC Xena database due to the lack of sequencing data for normal ovarian samples from TCGA. The “limma” package was used to identify CMRGs differentially expressed between OC and normal tissue. thresholds were set to FDR<0.05 and |log2(Fold change)| > 1.

### Consensus clustering analysis

We used the “ConsensusClusterPlus” package for cluster analysis by the k-means algorithm (19). Different CM clusters were identified based on the expression of CMRG. After 1000 tests to determine the appropriate number of clusters between k = 2-10 (20). The “limma” program was used to find differentially expressed genes (DEGs) in various CM clusters with FDR < 0.05 and |log2FC| > 0.5. A gene set variation analysis (GSVA) was carried out using “c2.cp.kegg.v7.2.symbols.gmt” extracted from the MSigDB database to look for variations in the biological processes of CM. The amount of immune cell infiltration in various clusters was assessed using the Single Sample Gene Set Enrichment Analysis (ssGSEA) technique (21). In addition, using the Kaplan R package generated by the “ survival “ and “survminer “ R packages generated Kaplan-Meier curves were used to assess the differences in OS between different clusters (22).

### Functional enrichment analysis

With the R package “cluster profile”, we performed GO enrichment analysis and KEGG signaling pathway analysis to investigate the possible biological roles and signaling pathways involved in these DEGs (23, 24). To investigate the differences in biological functions between the LR and HR groups, Gene Set Enrichment Analysis (GSEA) and Gene Set Variation Analysis (GSVA) were performed between the two groups. Downloaded from the MSigDB database and “c2.cp.kegg.v7.2.symbols.gmt” with thresholds set at P < 0.05 and FDR < 0.25 (25).

### Calculation of risk scores and construction of the CMRGPS

To calculate CM scores to quantify CM patterns in each sample. First, univariate Cox regression analysis was performed on the 544 DEGs associated with CM (p < 0.05) to identify the 40 DEGs associated with OC prognosis. second, a consensus clustering algorithm further classified OC patients based on the expression profiles of the 40 DEGs. The TCGA cohort was then utilized to compute the risk score associated with CM, with GSE63885 and GSE9891 serving as the validation group and TCGA serving as the training group. In short, the “glmnet” R package was utilized based on the prognostic genes connected to the CM clusters, and the Lasso Cox regression algorithm was applied to reduce the danger of overfitting. We examined the change cross-validation. To build a predictive signature for CM risk score-associated genes in the TCGA training set, candidate genes were chosen using multivariate Cox analysis.

The CM risk score was calculated as follows: CM risk score = Σ(Expi * coefi)

where coefi and Expi stand for each gene’s expression and risk factor, respectively (25). Patients in the TCGA training set were split into low and high-risk groups based on the median values, and Kaplan-Meier survival analysis was then performed on each group. Following that, the “ggplot2” R program was used to perform principal component analysis (PCA).

### Tumor microenvironment and tumor mutational load (TMB)

The tumor-infiltrating immune cells dataset is available for download at TIMER 2.0 (http://timer.cistrome.org). TIMER, CIBERSORT, quantTIseq, MCP-counter, xCELL, and EPIC algorithms were also compared (26). Single gene set enrichment analysis (ssGSEA) was used to score 28 immune cells from OC patients in the LR and HR groups. To identify somatic mutations in OC patients between the HR and LR groups, mutation annotation formats (MAF) from the TCGA database were generated using the “maftools” R package and we also calculated tumor mutation load (TMB) scores for each OC patient in both groups. Tumor purity and TME scores were estimated for each patient using the “estimate” package. Tumor Immune Single-Cell Hub (TISCH) is an extensive single-cell RNA-seq database dedicated to TME. It enables comprehensive analysis of TME heterogeneity across different datasets and cell types. We used a one-level logistic regression (OCLR) machine learning algorithm to quantify the stemness of tumor samples by calculating the tumor stem cell index (27).

### Immunotherapeutic response prediction and drug sensitivity assessment

We calculated the semi-inhibitory concentration (IC50) values of commonly used chemotherapeutic drugs for OC using the “pRRophetic” software package to examine the variations in the efficacy of chemotherapeutic medicines between the two groups of patients (28). TIDE, which stands for Tumour Immune Dysfunction and Rejection, is a computational framework for assessing the potential for tumor immune escape in the gene expression profile of tumor samples. The Immune Phenotype Score (IPS) is a valid predictor of response to immunotherapy targeting CTLA-4 and PD-1 (29). Tide and IPS were used to predict response to immunotherapy in both subgroups. Xu et al. created a website that offers us a collection of genes linked to cancer and immunology (30), as well as a list of genes favorably connected to Mariathasan’s research outcomes and anti-PD-L1 medication response (31).

### Immunohistochemical techniques and quantitative real-time polymerase chain reaction PCR (RT-qPCR)

Ovarian epithelial cell IOSE, ovarian cancer SKOV-3, and A2780 cell lines were obtained from the Shanghai Institutes for Life Sciences (Chinese Academy of Sciences, Shanghai, China) and maintained in Roswell Park Memorial Institute 1640 medium supplemented with 10% heat-inactivated fetal bovine serum, penicillin (10 U/mL) and streptomycin (50 µg/mL) at 37°C and 5% CO2 atmosphere. Total cellular RNA was isolated from cells and tissues using Trizol reagent (Invitrogen), and cDNA was obtained by reverse transcription using SuperScript II reverse transcriptase (Invitrogen) according to the manufacturer’s recommended protocol. next, SYBR Premix Ex Taq II (Takara, Dalian. China) to assess the relative mRNA expression levels of P2RY14 and GAPDH (as a normalized control). The primer sequences are as follows.

P2RY14:5”-TCTCACCAACCAGAGTGTTAGG-3”;5”-GCGCTAGATTTCTTTGACCG-3”.GAPDH:5”-GGAGCGAGATCCCTCCAAAAT-3”;

Transcriptomic and proteomic approaches were used to study protein expression at the RNA and protein levels in human tissues and organs, using data found in the Human Protein Atlas (HPA, https://www.proteinatlas.org/).

### Statistical analyses

All analyses were performed using R version 4.1.1, 64-bit 6, and its support package. In all statistical investigations, P<0.05 was considered statistically significant.

## Results

### Differential expression and genetic variation patterns of CMRGs in ovarian cancer

**Figure 1:**
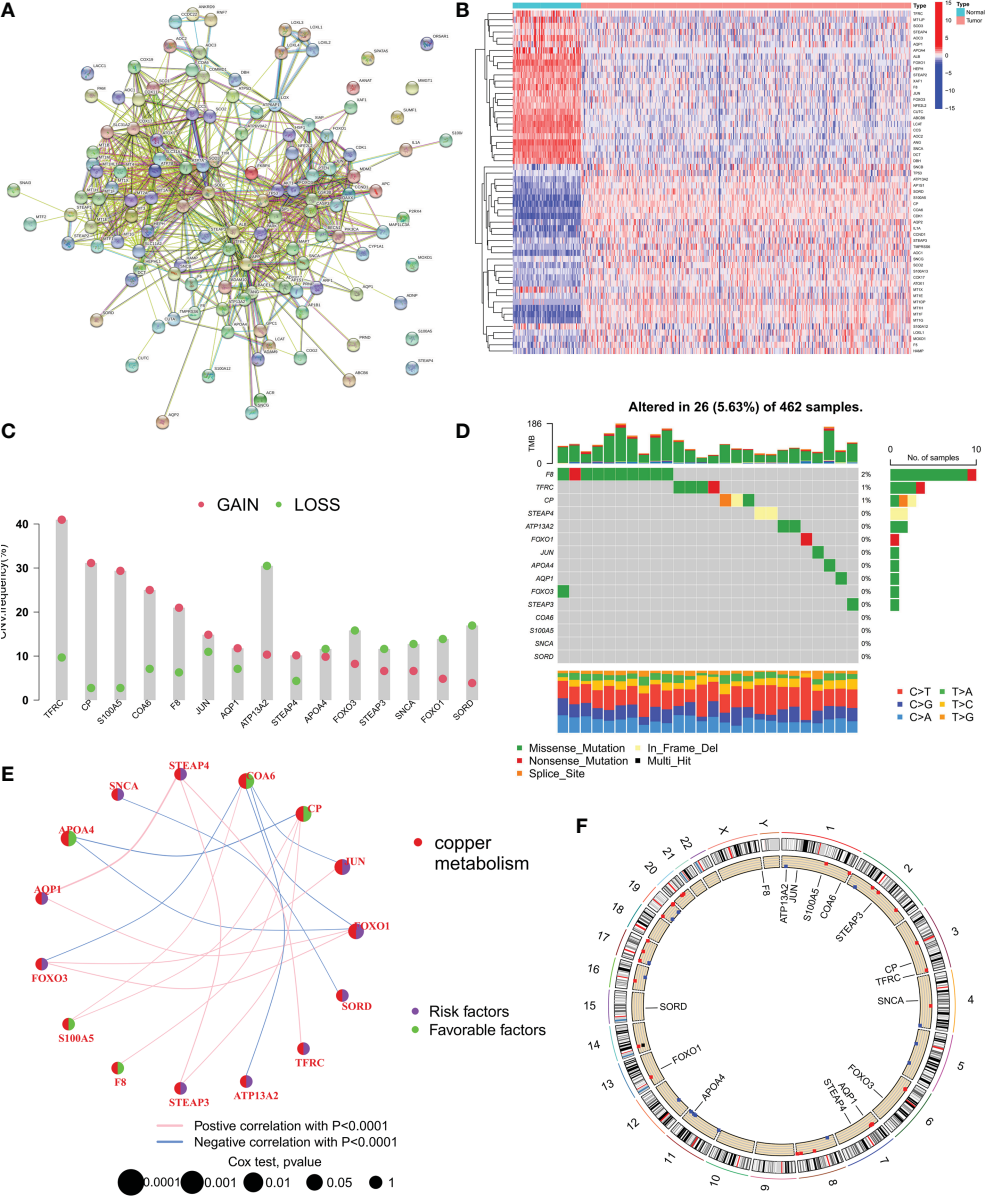
Expression and mutation of copper metabolism-related genes (CMRGs) in ovarian cancer. **(A)** Protein-protein interaction (PPI) network of all 133 copper metabolism-related genes (CMRGs). **(B)** Heat map of differential expression of CMRGs between tumor and normal tissues. **(C)** CNV frequencies of CMRGs in the TCGA cohort. **(D)** Mutation frequencies of 15 CMRGs in 462 OC patients in the TCGA cohort. **(E)** Network plot showing the correlation of 15 CMRGs in OC and the impact on prognosis. **(F)** The location of 15 CMRGs on 23 human chromosomes.

First, all 133 CMRGs were substituted into the String database, and protein-protein interaction network analysis revealed close associations between most CMRGs (**Figure 1A**). We performed differential expression analysis of 133 CMRGs in ovarian cancer and normal tissues and obtained 56 differentially expressed CMRGs (**Figure 1B**). Next univariate cox analysis (P<0.2) and Kaplan-Meier survival analysis (P<0.05) were used to select CMRGs that were prognostically significant for OC and 15 CMRGs were obtained (**Supplementary Table 2**). Next, we explored the level of somatic mutations and frequency of altered CNVs in the 15 CMRGs in ovarian cancer patients. The waterfall plot in **Figure 1D** shows that 26 of the 462 samples (5.63%) had mutations in the CMRGs. The highest frequency of F8 mutations was found (2%). Overall the frequency of mutations in CMRGs was extremely low. We also examined the frequency of altered CNVs in CMRGs and found that TFRC had the most significant copy number increase, while ATP13A2 had the most significant copy number deletion (**Figure 1C**). **Figure 1E** shows the interaction and prognostic impact of CMRGs in OC, suggesting a potential regulatory role of CNVs on the expression of CMRGs. Finally, **Figure 1F** shows the positioning of these CMRGs on the chromosome.

### Identification of CM clusters in OC

**Figure 2:**
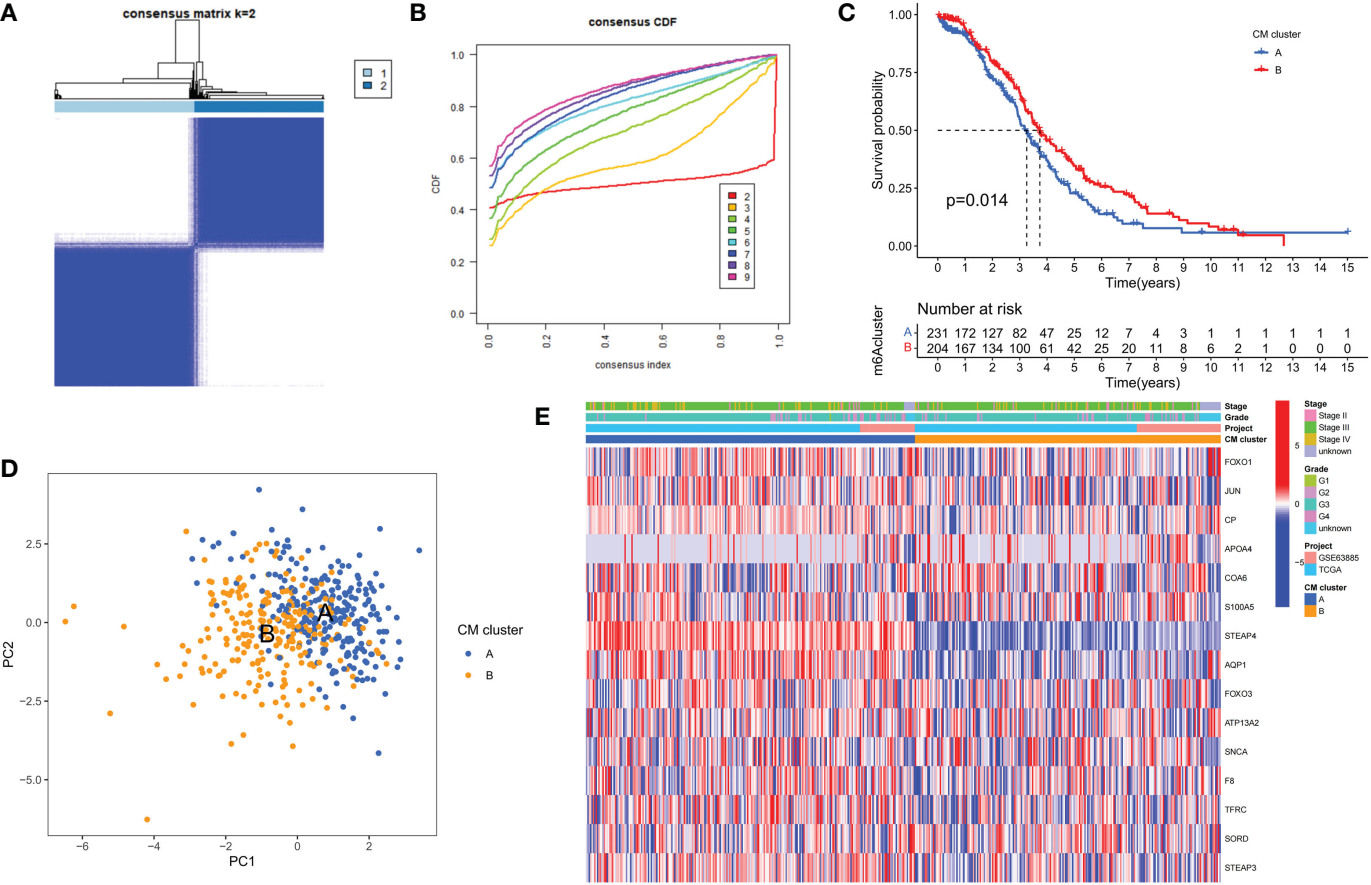
Clinicopathological and biological characteristics of the two CM clusters. **(A)** All samples from the TCGA-OV cohort and the GSE63885 cohort were divided into two clusters using a consensus clustering algorithm (k = 2). **(B)** The cumulative distribution function (CDF) from k = 2 to 9. **(C)** Kaplan-Meier curves show the different overall survival (OS) rates between the two CM clusters. **(D)** Principal component analysis (PCA) shows significant differences between the two CM clusters. **(E)** Heat map showing differences in clinical information and expression of CMRGs between the two clusters.

To fully understand the expression patterns of CMRGs involved in tumorigenesis, we integrated samples from the TCGA-OV and GSE63885 cohorts. To identify the different subtypes of OC, we used a consistent clustering algorithm and classified the samples according to the expression of 15 CMRGs (**Figure 2A**). the results of the CDF (Cumulative Distribution Function) curve showed that K=2 was the optimal number of clusters (**Figure 2B**). Therefore, the integration cohort was divided into CM clusters of 2 (**Supplementary Table 3**). Survival analysis showed that CM cluster B had a better OS (**Figure 2C**). Principal component analysis (PCA) confirmed a significant difference in the distribution of the two CM clusters (**Figure 2D**). In addition, we compared the expression of CMRGs and clinical information between the two CM clusters (**Figure 2E**).

**Figure 3:**
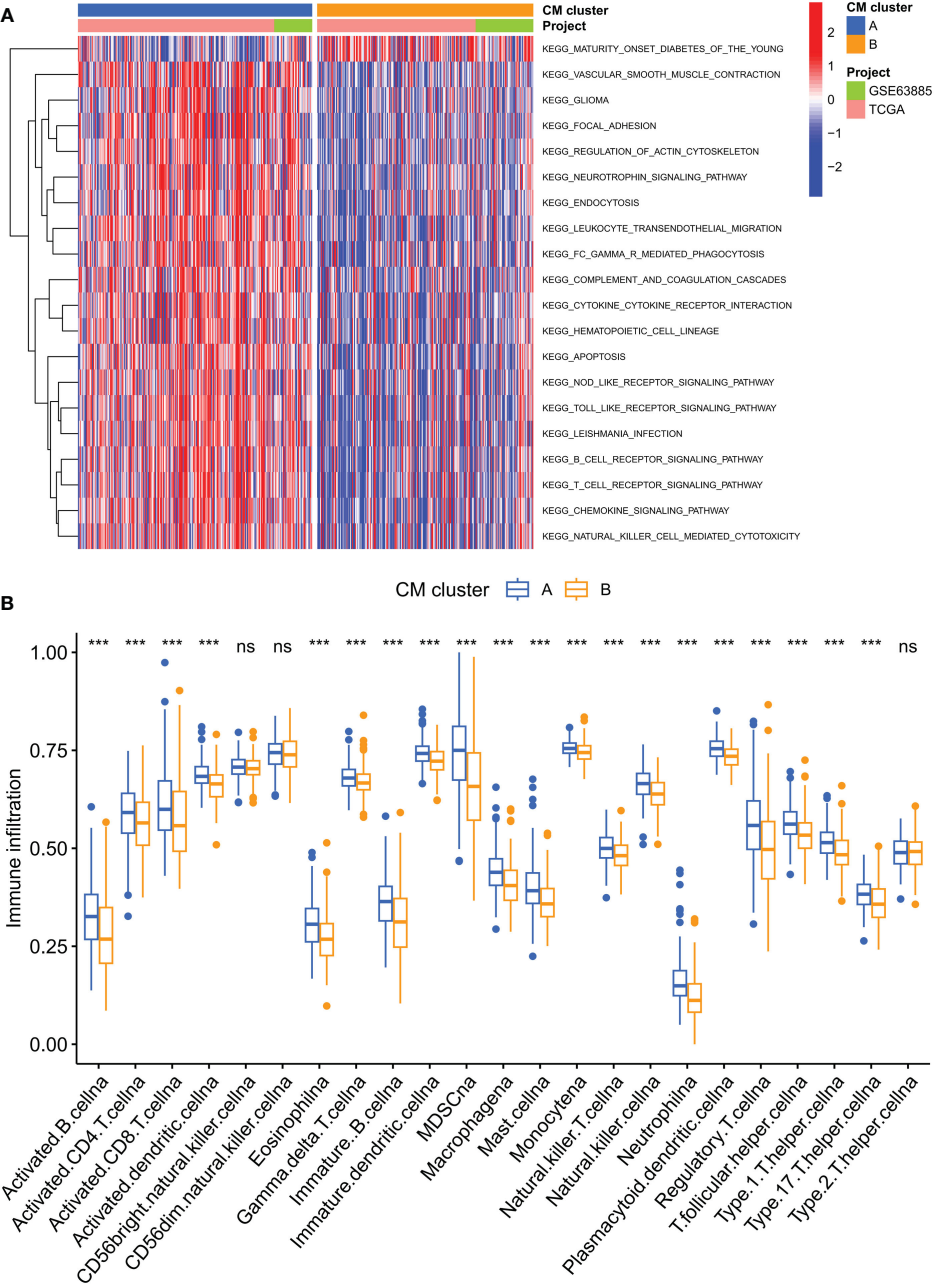
Analysis of the immune microenvironment of two CM clusters of tumors. **(A)** The abundance of 23 infiltrating immune cell species in two CM clusters. **(B)** GSVA of biological pathways between two clusters. ***p < 0.001; ns, no significance.

GSVA enrichment analysis showed that immune activation-related pathways were significantly enriched in cluster A, including Leukocyte transendothelial migration, Fc gamma R-mediated phagocytosis, immune cell receptor signaling pathways, cytokine receptor interactions and NOD-like and Toll-like receptor signaling pathways (**Figure 3A**). To investigate the role of CMRGs in TME, we evaluated the correlation between the two clusters and immune cell subpopulations separately using the ssGSEA algorithm. We observed significant differences in the infiltration of most immune cells between the two clusters. Compared to cluster B, cluster A possessed a higher immune cell infiltration, except for NK cells and T helper 2 cells (**Figure 3B**).

### Identification of CM gene clusters in OC

**Figure 4:**
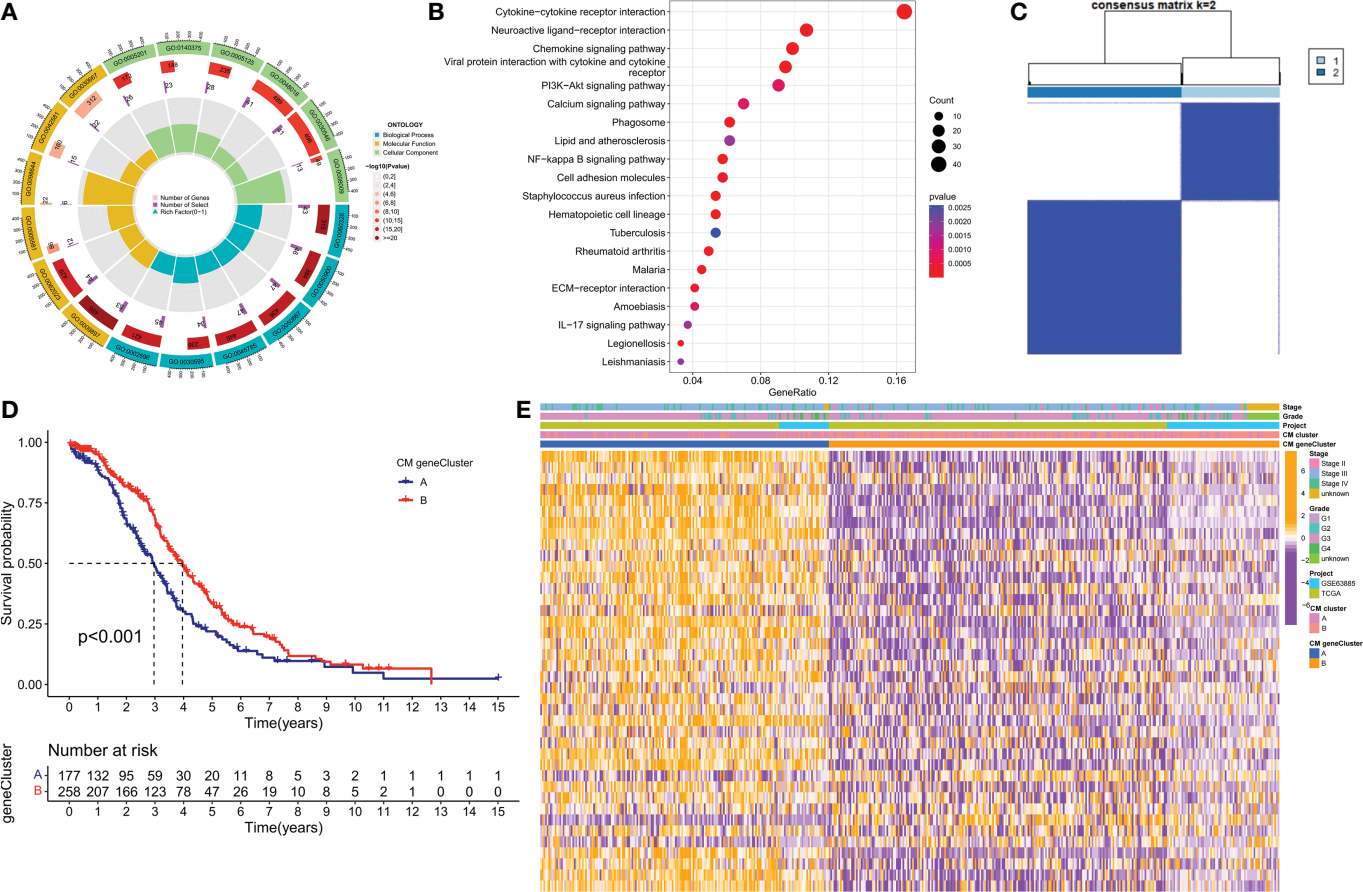
Identification of CM gene clusters based on differential genes (DEGs) in CM clusters. **(A, B)** GO and KEGG enrichment analysis of DEGs in two CM clusters. **(C)** All samples from the TCGA-OV cohort and the GSE63885 cohort were divided into two CM gene clusters using a consensus clustering algorithm (k = 2). **(D)** Kaplan-Meier survival analysis between different gene clusters. **(E)** Heat map of clinicopathological features and expression of DEGs. GO, Gene Ontology; KEGG, Kyoto Encyclopedia of Genes and Genomes.

To further investigate the potential biological behavior of each CM cluster, we identified 544 DEGs between the two CM clusters using the ‘limma’ package and performed a functional enrichment analysis of these DEGs. These genes were mainly involved in immune and cytokine-related pathways (**Figures 4A, B**). To determine the prognostic value of these DEGs, a univariate Cox analysis was performed to screen 40 DEGs for prognostic relevance, using a cut-off value of 0.05 as the p-value (**Supplementary Table 4**). Patients with OC were classified into 2 CM gene clusters using a consensus clustering algorithm (**Figure 4C** and **Supplementary Table 5**). Survival analysis showed that CM gene cluster B had a better prognosis (**Figure 4D**). Heat maps reflect differences in expression levels and clinicopathological factors of prognosis-related DEGs in the 2 CM clusters and the 2 CM gene clusters (**Figure 4E**).

### Construction and validation of the risk model

**Figure 5:**
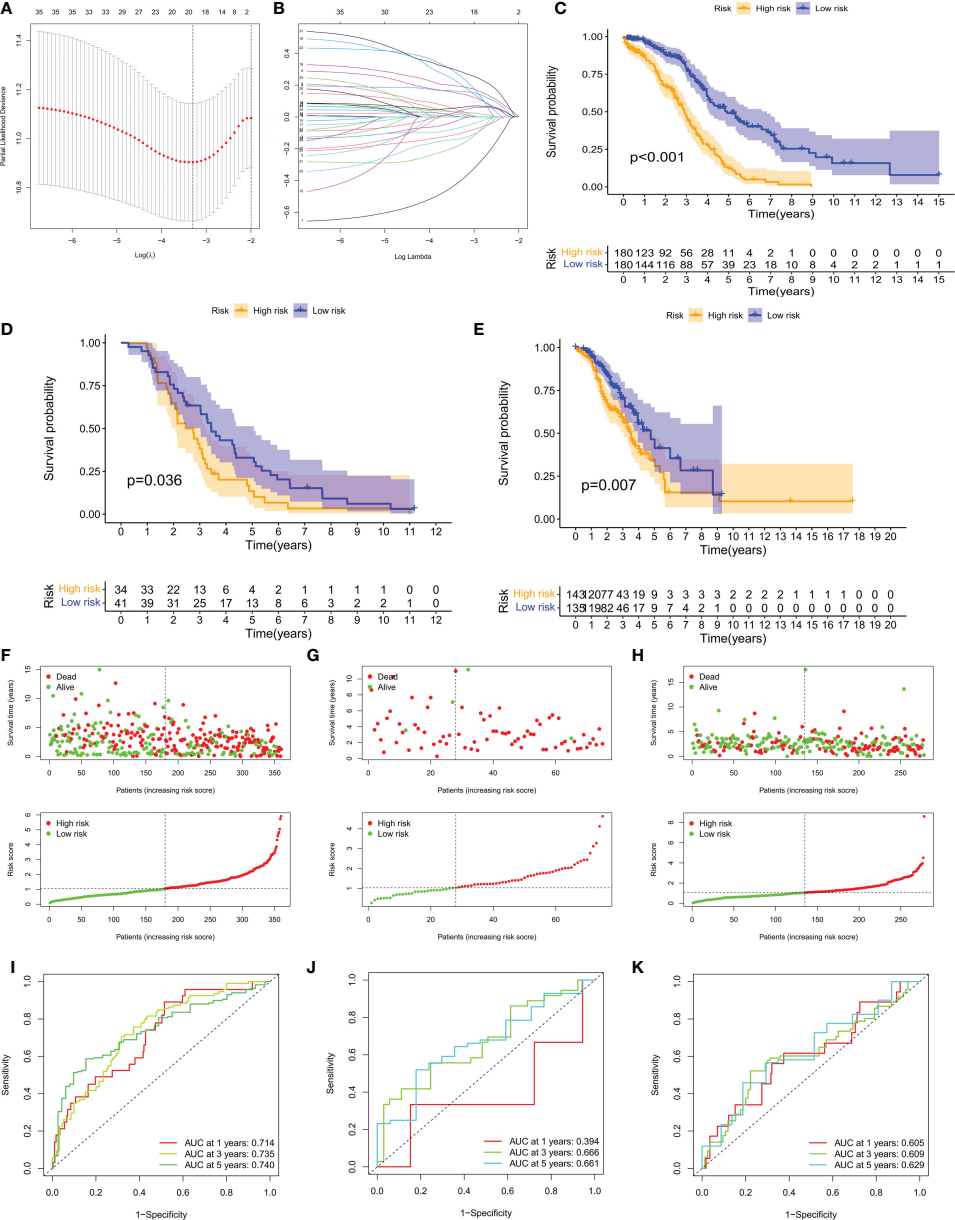
Construction of CMRGPS and prognostic value of risk scores. **(A, B)** Linear models (Lasso) were constructed and coefficients were visualized using LASSO Cox regression to identify 19 copper metabolism-related DEGs. **(C–E)** Kaplan-Meier survival curves showing the risk stratification ability of the TCGA-OV, GSE63885, and GSE9891 cohorts. **(F–H)** Risk plots were used to illustrate the survival status of each sample in the TCGA-OV, GSE63885, and GSE9891 cohorts. **(I–K)** AUC values for risk scores at 1, 3, and 5 years in the TCGA-OV, GSE63885, and GSE9891 cohorts.

A risk model based on 40 CM risk score-related DEGs was created to estimate the risk for each patient with ovarian cancer. First, in the TCGA training set, suitable risk models were built using LASSO and multivariate Cox regression analysis. Based on the least partial likelihood of deviance, LASSO regression analysis was used to screen 21 potential genes (**Figures 5A, B**). Multivariate Cox regression was then performed on the 21 prognosis-related genes, yielding 11 genes for use in constructing the risk model, namely RARRES1, ADH1B, LILRA2, TLL1, P2RY8, P2RY14, DHRS9, ZFHX4, CAMK1G, GPR171, and IL12A. We calculated the CM risk score for each patient based on the formula Risk score = (0.11 × RARRES1 expression) + (0.14 × ADH1B expression) + (0.25 × LILRA2 expression) + (0.24 × TLL1 expression) + (0.11 × DHRS9 expression) + (0.21 × ZFHX4 expression) + (0.14 × IL12A expression) - (0.32 × P2RY8 expression) - (0.49× P2RY14 expression) - (0.21× CAMK1G expression) - (0.24× GPR171 expression). Patients were divided into high-risk (HR) and low-risk (LR) groups based on the median value of the CM risk score for the TCGA training cohort. Notably, the LR group in the TCGA cohort had a higher overall survival (OS) rate than the HR group (p < 0.001, **Figure 5C**). For the GSE63885 and GSE9891 validation cohorts, patients in the LR group also had better OS (**Figures 5D, E**). risk plots for the TCGA-OV, GSE63885 and GSE9891 cohorts also showed specific survival outcomes for each patient, with patients in the HR group typically having poorer survival outcomes (**Figures 5F–H**). Strikingly, in the TCGA cohort, our constructed CMRGPS performed very well in predicting OS in these patients, with AUCs of 0.7 at 1, 3, and 5 years (**Figure 5I**). the predictive power of CMRGPS was also validated in the GEO cohort (**Figures 5J, K**).

### Validation of prognostic signatures of genes related to copper metabolism and construction of nomograms

**Figure 6:**
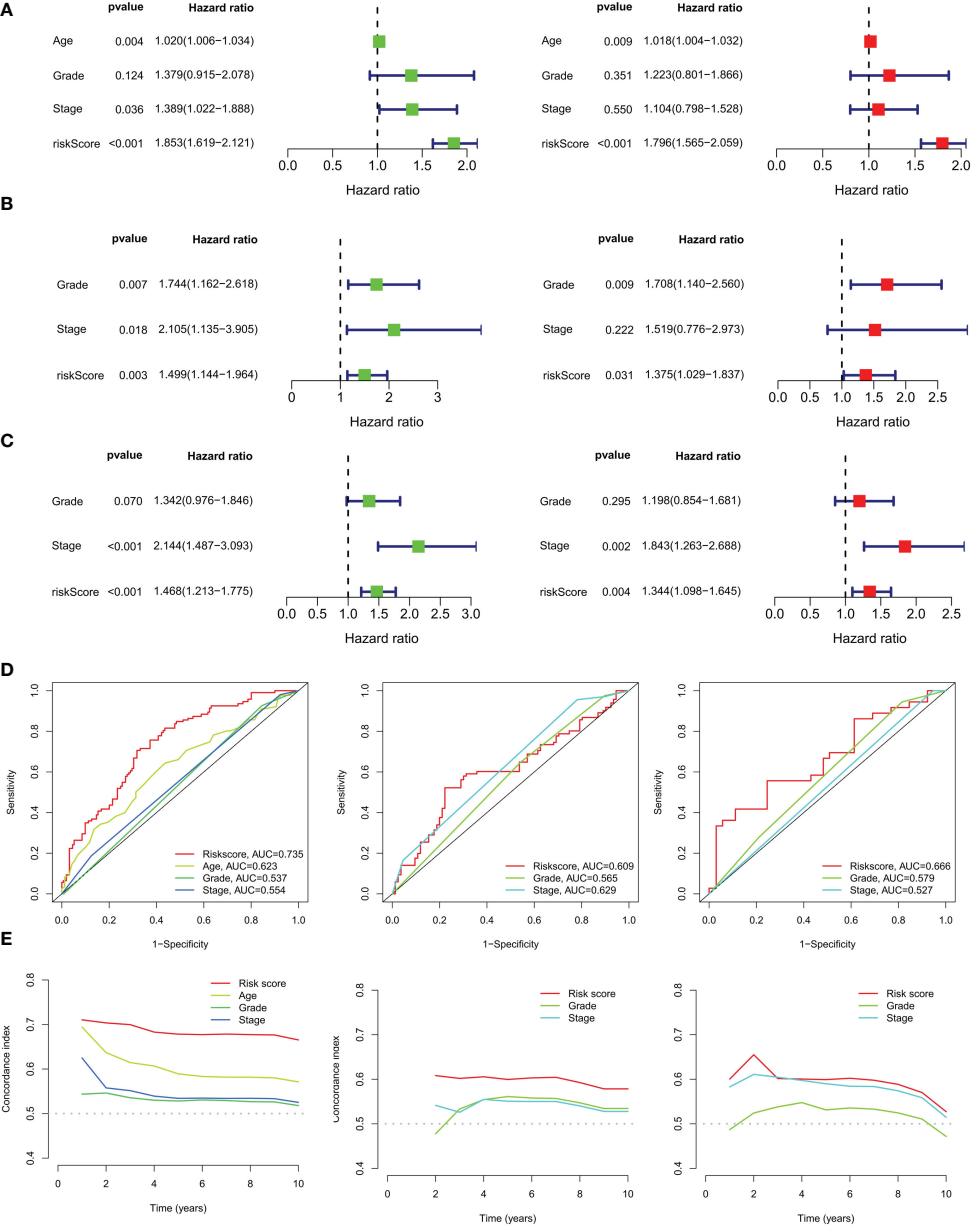
Independent prognostic analysis of ovarian cancer risk scores and clinicopathological factors. **(A)** Univariate and multivariate Cox regression analyses of clinicopathological variables and risk scores in the TCGA training cohort, **(B)** GSE63885 and **(C)** GSE9891 validation cohorts. **(D)** AUC values for risk scores and clinical characteristics of TCGA-OV, GSE63885, and GSE9891 at 3 years. **(E)** Coherence index (C-index) for the TCGA-OV, GSE63885, and GSE9891 cohorts.

In the TCGA training set, CM scores can be an independent prognostic indicator for patients compared to other common clinical characteristics (age, grade, stage) based on the results of univariate and multivariate Cox analyses (**Figure 6A**). Our constructed CMRGPS was also validated in the GSE63885 and GSE9891 cohorts (**Figures 6B, C**). In addition to this, the area under the curve (AUC) of the CM score at three years was much higher than other clinicopathological features (**Figure 6D**). The C-index of the CM risk score was also much greater than that of the other clinical features (**Figure 6E**).

**Figure 7:**
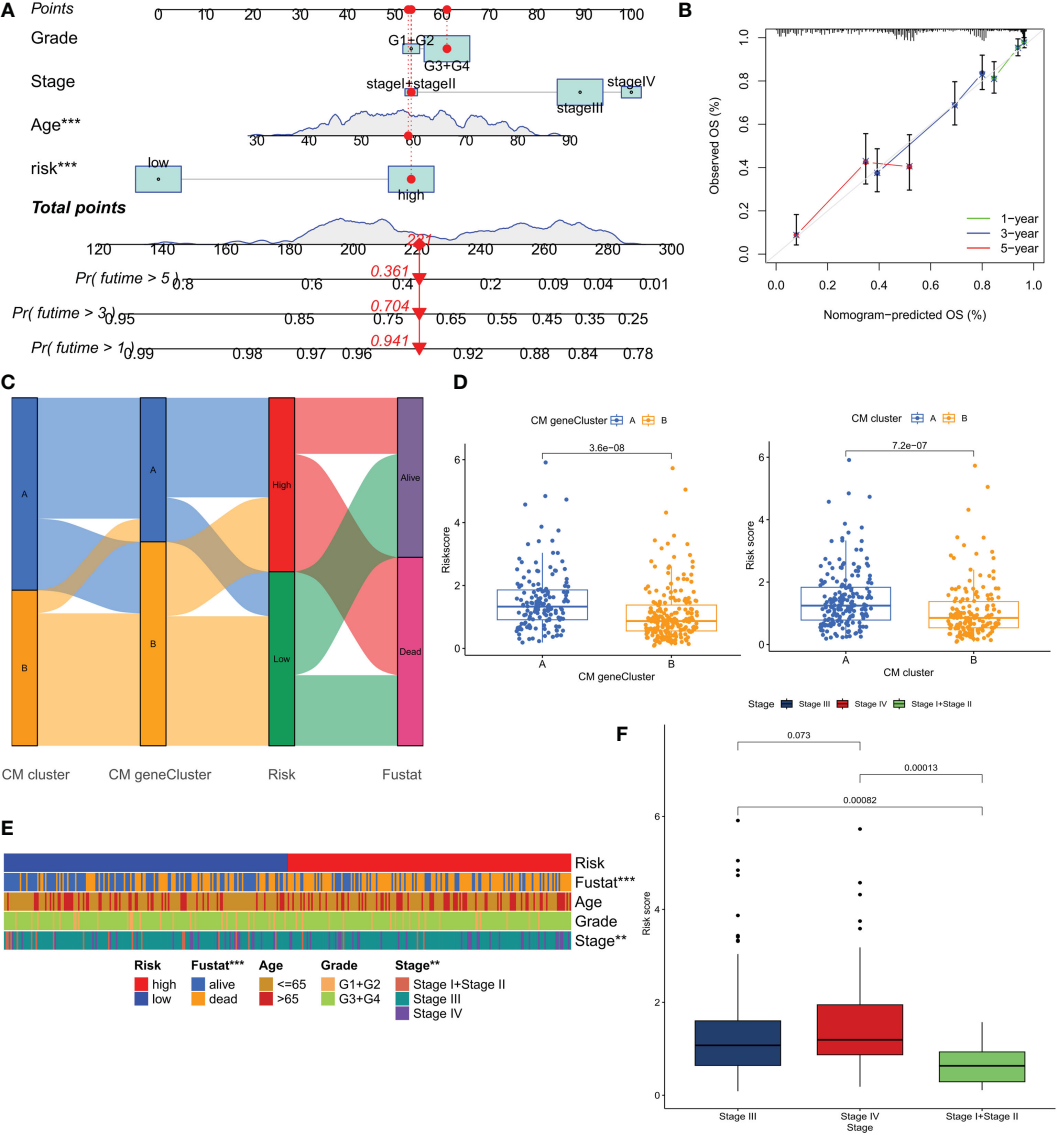
Construction and validation of nomograms for predicting OS in ovarian cancer in the TCGA cohort. **(A, B)** Combined nomograms and calibration curves for age, grade, and stage were used to predict OS at 1, 3, and 5 years in ovarian cancer patients. **(C)** Alluvial plots show the distribution of patients in 2 CM clusters, 2 CM gene clusters, 2 risk groups, and their survival status. **(D)** Differences in risk scores for the 2 CM clusters and the 2 CM gene clusters. **(E)** Heat map of clinical characteristics associated with risk clusters as determined by chi-square test. **(F)** Comparison of risk scores of patients at different clinical stages. **P < 0.01; ***P < 0.001.

Based on the above correlation between clinicopathological features and CM risk scores, a nomogram was created to predict survival at 1, 3, and 5 years for patients with OC (**Figure 7A**). The calibration curve showed that the nomogram was able to make accurate predictions (**Figure 7B**). The Alluvial plot showed that the CM cluster B and CM gene cluster B with better prognosis mostly corresponded to the LR group (**Figure 7C**). And these two groups also had lower CM scores (**Figure 7D**). The results of the chi-square test showed that the risk grouping was only related to the survival status and tumor stage of the patients (**Figure 7E**). **Figure 7F** shows that stage IV patients had a higher CM score. Based on the results of the above analysis, we are more confident that CMRGPS is a reliable clinical prediction tool.

### TMB analysis and survival analysis of TMB

Additional evidence suggests that patients with high TMB may benefit from immunotherapy due to higher antigen counts (32). We generated two waterfall plots to explore the detailed gene mutation profiles between the LR and HR group populations (**Supplementary Figures 1A, B**). We found that TP53 and TTN were the most commonly mutated genes in OC, with no significant differences in mutation profiles between the two groups. Different mutational status and expression patterns may lead to different clinical outcomes of the immune response. TMB analysis showed no significant difference between the two groups (p>0.05), with a higher TMB in the LR group (**Supplementary Figure 1C**). Survival analysis was performed by dividing the patients into high and low TMB groups based on the median TMB values obtained, and the combined application of CM score and TMB to divide the patients into four subgroups for survival assessment showed that the high TMB and LR groups had the best prognosis (P<0.001), which helped to screen the best prognostic subgroups for clinical use (**Supplementary Figure 1D**).

### Tumor microenvironment (TME) and immune cell infiltration

**Figure 8:**
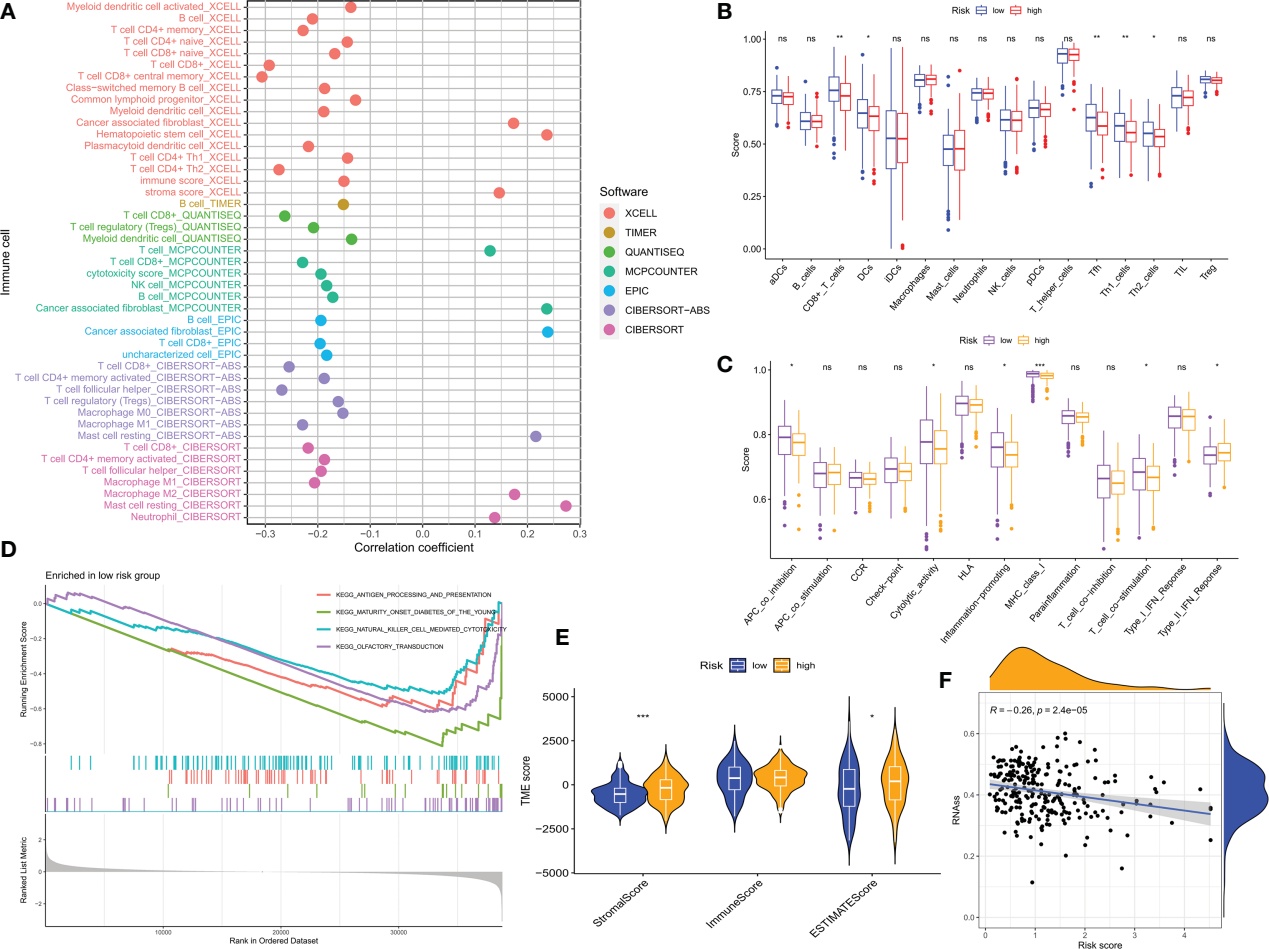
CM risk score predicts TME and immune cell infiltration. **(A)** Bubble plots obtained by different algorithms show the correlation between risk scores and immune cell content. **(B)** Differences in immune cell infiltration between populations in different risk groups. **(C)** Differences in immune function between populations in different risk groups. **(D)** CMRGPS-based enrichment analysis of KEGG gene sets in low-risk populations. **(E)** Differences in TME scores between populations in different risk groups. **(F)** Correlation of cancer stem cell index (RNAss) with risk scores. *p < 0.05, **p < 0.01, ***p < 0.001; ns, no significance.

The tumor microenvironment (TME) influences the clinical outcome of patients and their response to treatment. Among these, tumor-infiltrating immune cells (TIICs) have a significant impact on tumor development and the efficacy of anti-tumor therapy. Although TIICs are an important component of TME, their composition and distribution are closely related to tumourigenesis and progression (33). Therefore, we investigated the correlation between CM scores and tumor immune cells based on various algorithms (**Figure 8A**), with lower CM scores correlating with the degree of T-cell infiltration. Enrichment scores for various immune cell subpopulations, related activities, or pathways were measured using the ‘ssGSEA’ method to further investigate the relationship between CM scores and immune cells and function. The results of the study showed that the LR group had higher scores for immune-related function and immune cell infiltration (**Figures 8B, C**). Based on the better prognosis and level of immune infiltration of patients in the LR group, a GSEA analysis was performed to explore the potential biological functions of the LR group. Based on normalized enrichment scores (NES) and P values, we selected the four most important enriched signaling pathways (**Figure 8D**), with lower CM scores associated with immune-related signaling pathways. The tumor stem cell index is an index to assess the similarity of tumor cells to stem cells and is associated with biological processes active in tumor cells (34). Therefore, we assessed the correlation between the RNA stemness score (RNAss) and the CM risks score. The results showed a significant negative correlation between CM score and RNAss (**Figure 8F**), indicating that OC cells with lower CM scores had more prominent stem cell characteristics and lower levels of cell differentiation.

**Figure 9:**
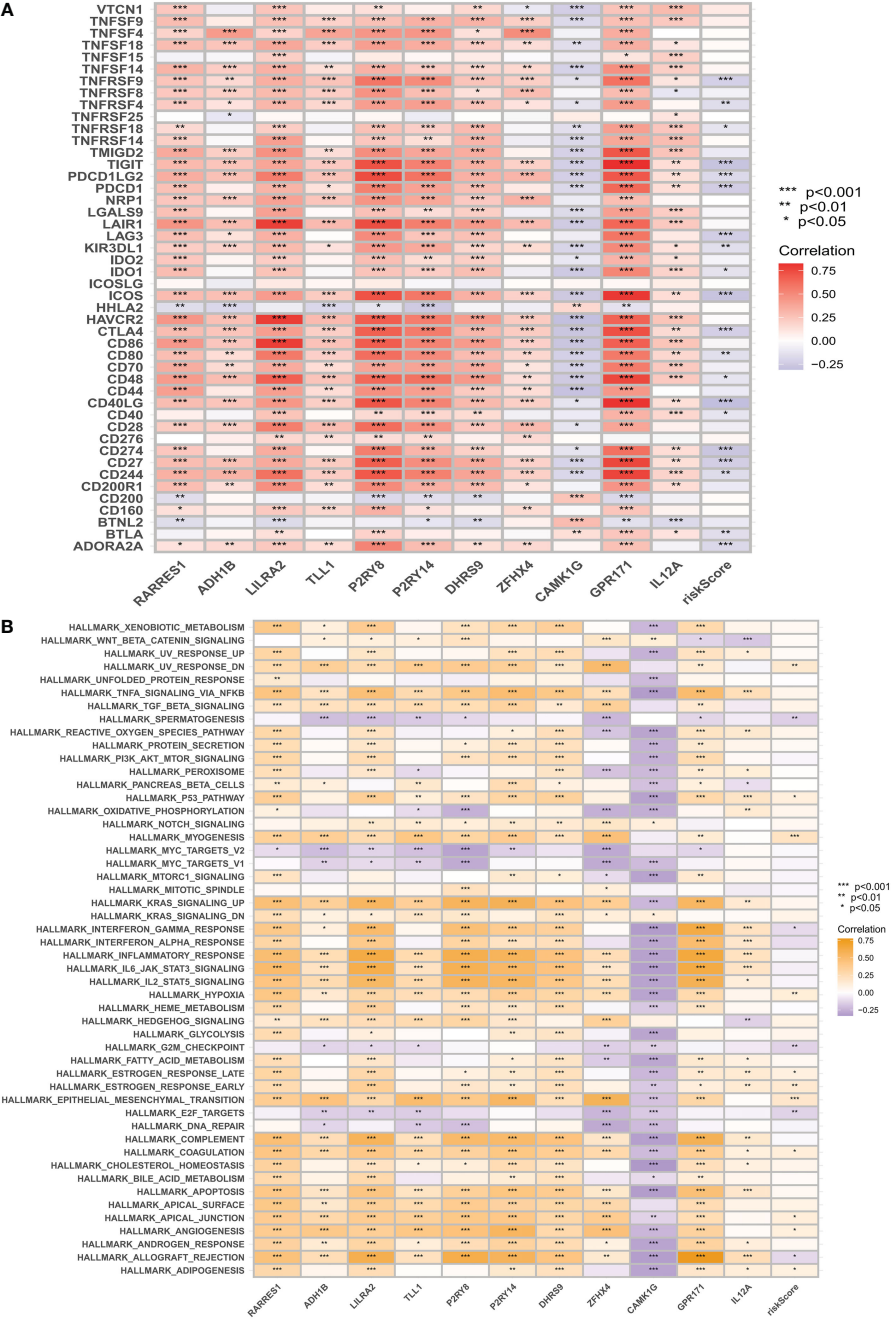
Immune checkpoint correlation analysis and GSVA correlation analysis. **(A)** Correlation of expression of all immune checkpoints with risk score-related genes and risk scores. **(B)** GSVA was used to analyze the correlation between the MiSigDB Hallmark pathway and risk scores. *p < 0.05, **p < 0.01, ***p < 0.001.

Due to the significant impact of abnormal expression and function of immune checkpoint molecules on tumor immunotherapy, we assessed the correlation between CM scores and expression of immune checkpoints (ICs). In particular, almost all immune check genes and our risk score-related genes showed an extremely strong correlation. Overall, our CM scores were negatively correlated with the expression of immune checkpoints such as PD1 (**Figure 9A**). the GSVA results showed that the risk score-related genes were associated with the hallmark pathway and that HR patients were associated with epithelial-mesenchymal transition (EMT) (**Figure 9B**). Thereafter, we used ESTIMATE to calculate the proportion of stromal and immune cells in the different risk groups to estimate tumor purity (**Figure 8E**), with higher stromal scores in the HR group. These findings suggest that patients in the LR group have a better prognosis, are more immune, and may be more sensitive to immunotherapy.

### Prediction of response to immunotherapy based on CMRGPS

**Figure 10:**
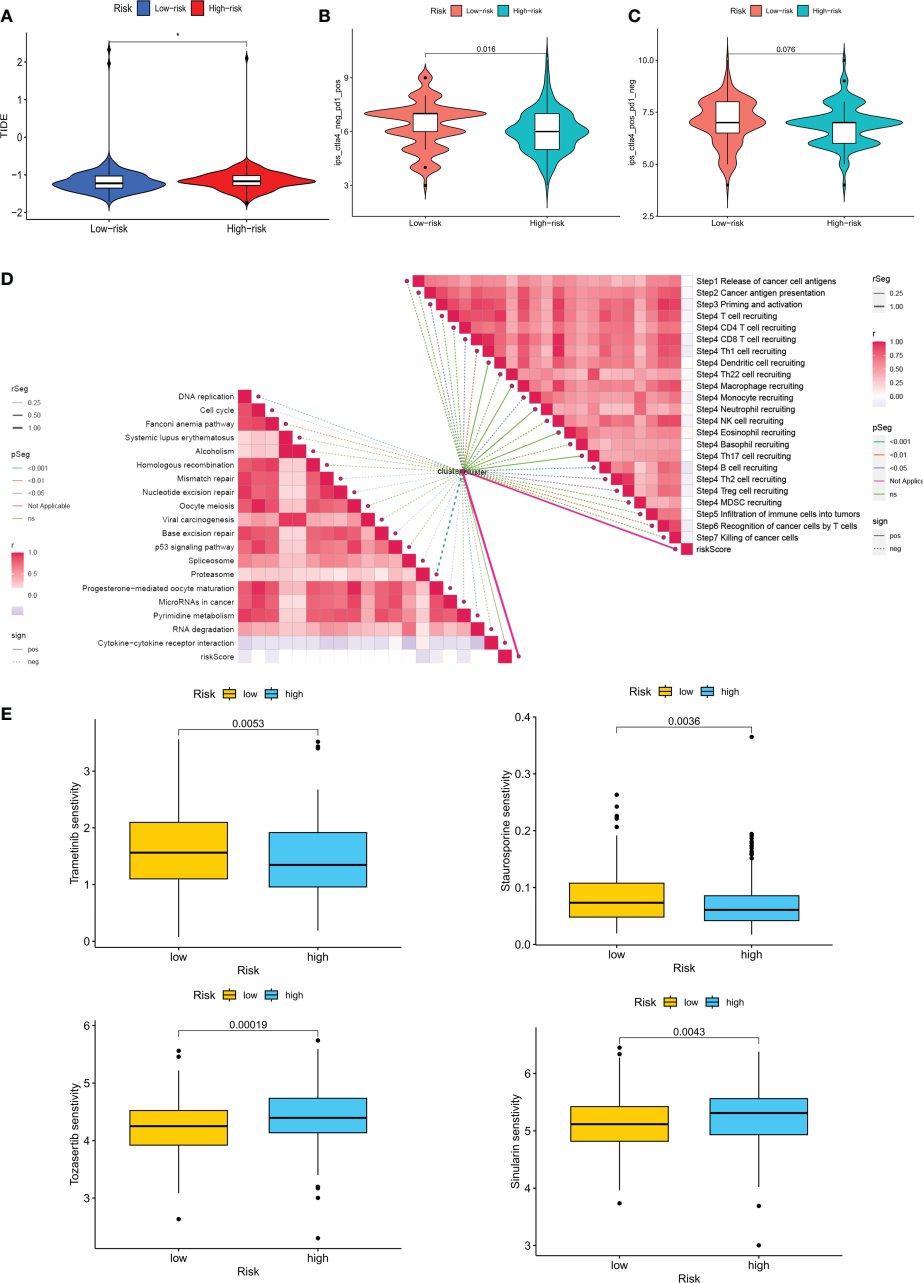
Analysis of treatment outcomes between the high-risk and low-risk groups. **(A)** TIDE analysis between the high-risk and low-risk groups. **(B, C)** Comparison of the relative distribution of immune scores (IPS) between the high-risk and low-risk groups. **(D)** Correlation of risk scores with ICB response characteristics and each step of the tumor-immune cycle. **(E)** Sensitivity to Tozasertib, Staurosporine, Trametinib, and Sinularin in the high-risk and low-risk groups. *p < 0.05.

To rationalize the selection of which patients are more suitable for immunotherapy, we applied the TIDE score to assess possible abnormalities in the immune function of the tumor and regional lymph nodes. The results showed that patients in the LR group had a higher probability of responding to immunotherapy (**Figure 10A**). In addition, IPS scores showed that the LR group responded better to treatment with PD1 inhibitors compared to CTLA4 inhibitors (**Figures 10B, C**). Immune checkpoint blockers (ICB) are the most well-studied class of immunotherapeutic agents that block inhibitory signaling of T-cell activation, enabling tumor-reactive T cells to mount an effective anti-tumor response (35). However, ICB therapy is only effective in a subset of patients. To further explore the role of CM scores in immunotherapy, we explored the correlation between CM scores and signals associated with ICB. The results showed that CM scores were negatively correlated with some signals such as the proteasome, Fanconi anemia pathway, p53 signaling pathway, and Pyrimidine metabolism (**Figure 10D**). Similarly, we investigated a significant negative correlation between each step in the tumor immune cycle, such as excitation and activation (step 3), and CM score (**Figure 10D**). The above results suggest that patients in the LR group may respond better to ICB treatment. Subsequently, we introduced four chemotherapeutic agents in the present study. We found that patients in the HR group were more sensitive to Trametinib and Sinularin. In contrast, patients in the LR group were more sensitive to Tozasertib and Staurosporine (**Figure 10E**).

### Validation of CM risk score-related genes

**Figure 11:**
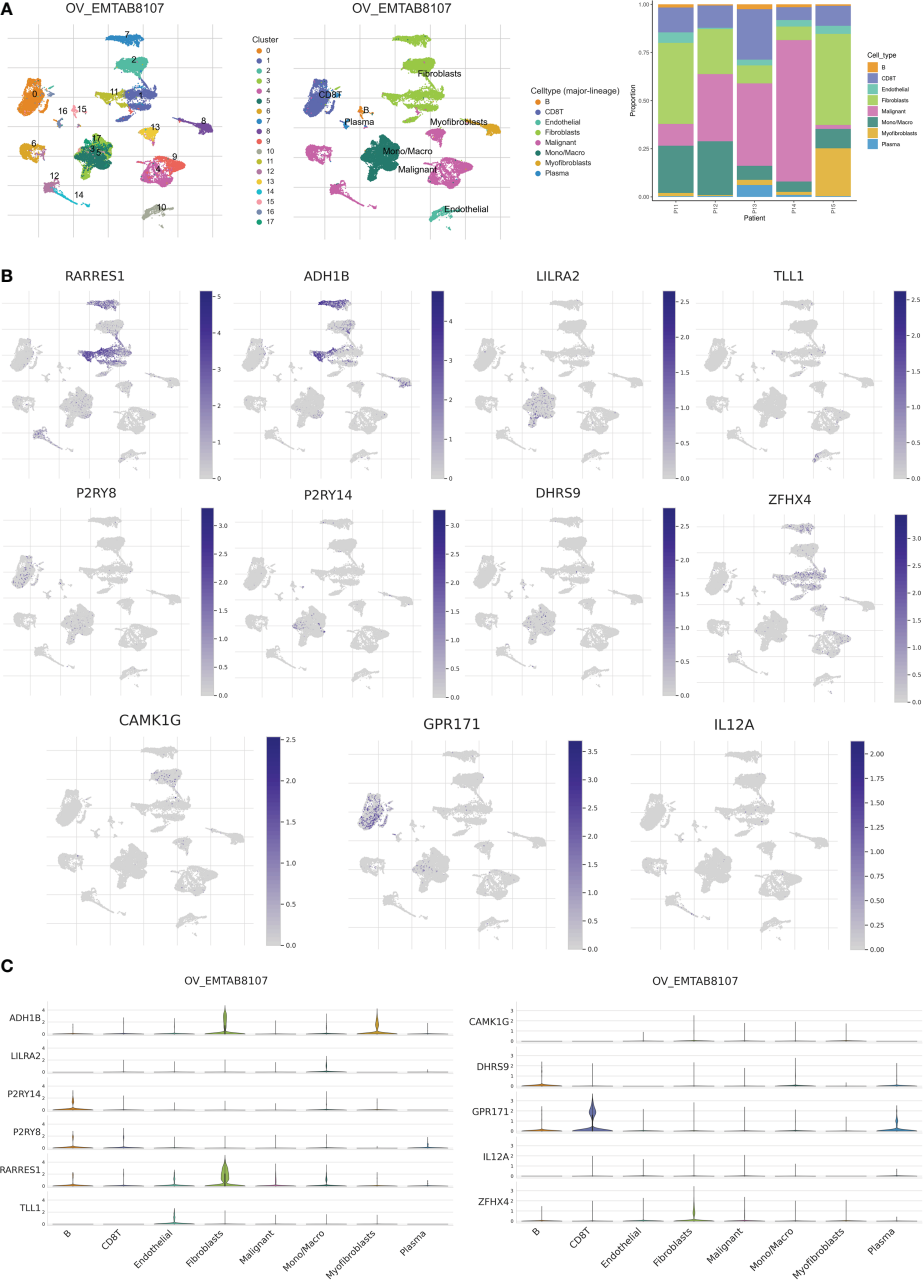
Validation of single-cell RNA sequencing. **(A)** Annotation of all cell types in EMTAB8107 and percentage of each cell type. **(B, C)** Expression of RARRES1, ADH1B, LILRA2, TLL1, P2RY8, P2RY14, DHRS9, ZFHX4, CAMK1G, GPR171 and IL12A in each cell type.

To analyze the expression of 11 genes associated with risk score in TME, we used the ovarian cancer single-cell dataset EMTAB8107 from the TISCH database. The EMTAB8107 dataset contains 8 major cell types and 18 major cell populations. **Figure 11A** displays the distribution and number of the various cell types. ADH18 and RARRES1 are mainly expressed in fibroblasts, and GPR171 is mainly expressed in CD8 T cells (**Figures 11B, C**).

**Figure 12:**
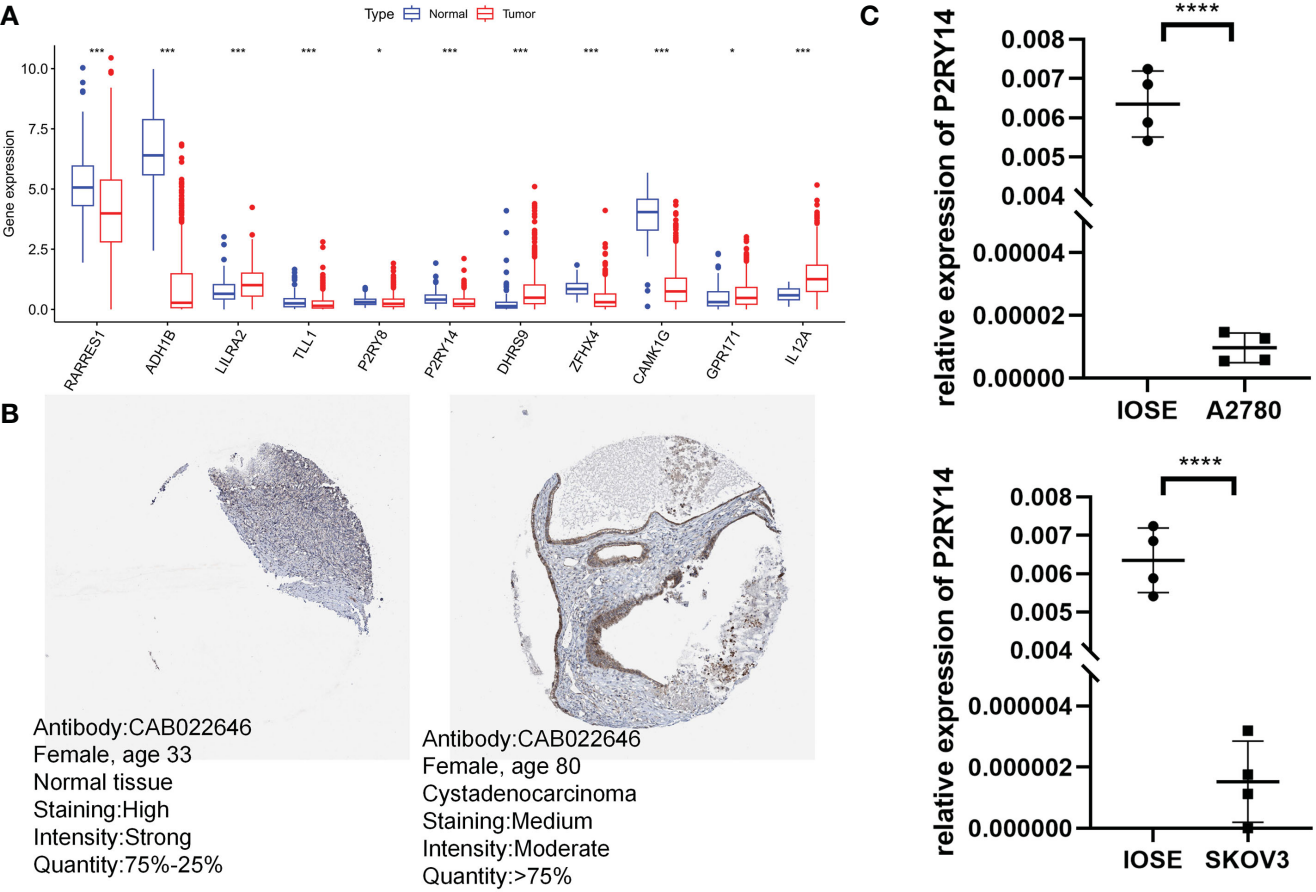
Validation of risk score-related gene expression. **(A)** Eleven genes show differences in normal and tumor cohorts. **(B)** Immunohistochemical analysis of P2RY14 in normal ovarian tissue and ovarian cancer. **(C)** qRT-PCR analysis of P2RY14. *P < 0.05; ***P < 0.001; ****P < 0.001.

Surprisingly, 11 risk score-related genes were differentially expressed in both the normal and tumor groups of the TCGA-GTEx cohort (**Figure 12A**). To validate the expression pattern of risk score-related genes in OC patients, we explored immunohistochemical data from the HPA database. Comparing ovarian cancer tissues with healthy ovarian tissues, P2RY14 protein expression levels were much higher in normal tissues (**Figure 12B**). Using qRT-PCR, we also found that P2RY14 was expressed at lower levels in ovarian cancer cell lines relative to normal cells (**Figure 12C**). Therefore, we hypothesized that aberrant expression of these genes may promote the malignant transformation of ovarian cancer.

## Discussion

Copper is an important trace element in the body, and the processes of uptake, transport, storage, and excretion of copper ions together determine the regulation of copper metabolic homeostasis, and both excess and deficiency of copper ions can lead to various diseases (36). The degree of dependence of tumor cells on mitochondrial metabolism determines the sensitivity of the cells to copper ions. The lack of copper metabolism-related proteins leads to the accumulation of copper ions in tumor cells, resulting in tumor resistance to radiotherapy (37). Many studies in recent years have pointed out that disorders of copper metabolism can promote tumor development by activating tumor proliferation-related signaling pathways, regulating tumour micro angiogenesis, and remodeling the stromal and inflammatory microenvironment. Elevated copper levels in cancer cells and serum copper cyanobactin were observed in patients with advanced ovarian cancer (38). In addition, copper transporter protein expression was associated with platinum resistance in ovarian cancer (39). Moreover, the copper-transporting ATPases ATP7A and ATP7B have been shown to regulate drug resistance in ovarian cancer (40). Therefore, this study aimed to reveal the immune profiles of different CM-associated clusters and delve into the prognostic value of CMRG in OC, to find potential targets for immunotherapy and to provide a protocol for precise and personalized treatment of OC patients.

In this work, we first used the TCGA-OV dataset to examine the differential expression levels and genetic mutation features of 133 CMRGs. We discovered two unique CM clusters, CM Cluster A and CM Cluster B, based on an unsupervised clustering technique of the transcriptome expression levels of CMRGs. Despite having less immune infiltration, individuals with OC in CM Cluster B had a better prognosis than those in Cluster A. Then, using the 40 DEGs found between the two distinct CM clusters, we found 2 gene clusters. The OS difference between the two gene categories was statistically significant. This reveals a strong relationship between CM clusters and gene clusters. We then constructed prognostic models of CM risk score-associated DEGs by lasso-cox, including RARRES1, ADH1B, LILRA2, TLL1, P2RY8, P2RY14, DHRS9, ZFHX4, CAMK1G, GPR171, and IL12A.

Together, these genes constitute a stable OC CM score profile. The results of the differential analysis showed that all 11 genes differed in tumor and normal tissues, and we finally selected P2RY14 for experimental study.P2RY14 is thought to be potentially associated with immune invasion in lung cancer and plays an important role in suppressing immune escape of tumor cells within the lung cancer microenvironment (41). In head and neck cancer, P2RY14 is also a potential biomarker for immune regulation of the tumor microenvironment and good prognosis (42). In addition, more meta-analyses have shown that potentially functional polymorphisms of IL12A and IL12B are thought to increase the risk of malignancies such as gastric, lung, and cervical cancers (43–45). TLL1 is significantly upregulated in OC patients and has been suggested as a prognostic marker in OC patients (46, 47), and the same results were obtained in our study. Zou et al. suggested that RARRES1 may induce autophagy in prostate and cervical cancer cells. And RARRES1 contributes to the regulation of dendritic cells and serves as a novel immune-related biomarker for glioblastoma (48). Several other bioinformatic analyses have pointed to the close association of ADH1B with the prognosis of ovarian cancer patients and that ADH1B is a potential source of chemoresistance in ovarian cancer (49, 50). ADH1B was also discovered to dramatically upregulate tumor cell adhesion and cell spreading, suggesting that it could improve the mesothelial clearance of ovarian cancer (50). The majority of the genes in our model have the potential to affect the course and prognosis of OC. In the training and validation sets, we classified OC patients into HR and LR groups based on the CM risk score, and we found that patients in the LR group had a considerably better prognosis than those in the HR group. The CM risk score was demonstrated to be a standalone prognostic predictor for OC by multi-factor cox regression. To extend the value of the CM risk score in clinical practice, a nomogram was constructed by combining common clinical indicators to provide clinicians with a personalized prognostic risk scoring system to personalize treatment for patients.

Ovarian cancer responds poorly to immunotherapy. Nevertheless, determining the sensitivity of specific treatment subgroups based on tumor biomarker stratification may increase the prediction of immunotherapy response. TMB, PD-L1, TIICs, and neoantigens in intra-tumor heterogeneity are some of these indicators. The use of these biomarkers to choose the best candidates for ovarian cancer treatment is one of the future directions (51).

There is a consensus on the important influence of the tumor microenvironment on various tumor phenotypes. One of the primary immunological characteristics of the tumor microenvironment is immune cell infiltration, which is crucial for the immune evasion of tumor cells and the development of an inflammatory environment (52, 53). We investigated the relationship between CMRGPS and the degree of immune cell infiltration as a result. We discovered that T cells had a stronger negative connection with a risk score, indicating higher levels of T cell infiltration in the LR group. Due to the immunological activation of TME, the LR group had a better prognosis and immunotherapeutic response, as was expected. Immune checkpoints serve as immunological system controllers and are crucial for preserving autoimmune tolerance as well as controlling the intensity and duration of immune responses in peripheral tissues (54, 55). We investigated the association between immunological checkpoints and risk scores and found that CTLA4 and PD1 showed a significant negative correlation with risk scores. While the presence of PD-L1 molecules on the membrane surface contributes to the suppression of T-cell activity, the expression of PD-L1 molecules in the cytoplasm of ovarian cancer cells is functional and supports the proliferation and invasion of tumor cells. Both PD-L1 and PD-1 monoclonal antibodies were used to exert anti-tumor effects in ovarian cancer models (56, 57). In addition, PD-1 molecules can further mediate immune escape through tumor-associated cells, and tumor-associated macrophages (TAMs) characterized by the expression of PD-1 molecules are important in the development of the disease (58). Finally, our analysis by TIDE and IPS scoring systems also reinforced the above results that LR patients are more suitable for immunotherapy.

Therefore, we evaluated the correlation between several clinically used drugs and risk scores. We found that LR patients were more sensitive to chemotherapy with Tozasertib, a pan-Aurora inhibitor that exhibited enhanced carboplatin activity in platinum-sensitive and platinum-resistant ovarian cells of different p53 statuses. At low doses, the compound synergized paclitaxel-induced apoptosis and was active against paclitaxel-resistant cells (59). A phase I trial of 24-hour continuous intravenous volasertib in 27 patients determined that the disease was stabilized in almost half of the patients (60). Thus, our study also gives clinicians a protocol to accurately screen patients for characteristics and a new perspective on clinical antineoplastic drug combination strategies.

Although the risk scoring system we have constructed is outstanding in its ability to identify the immune microenvironment of patients and to predict their prognosis. However, several limitations still require us to acknowledge and find appropriate ways to address them in subsequent studies. Firstly, the TCGA-OV dataset we included was predominantly white, and more data from other ethnic groups will subsequently need to be collected for validation. Secondly, more data from OC patients need to be collected to validate the utility of the model and the accuracy of immunotherapy predictions. In addition, more ex vivo experiments are needed to refine the relevant details of this study.

## Conclusions

Metabolic disorders are an important feature of malignant tumors. In recent years, the important role of copper metabolism in the evolution of tumors has come to the fore. Bioinformatics studies on copper metabolism will be a popular research direction in the future. As a result, we have shown for the first time that CMRGPS is a distinct predictive biomarker and potential therapeutic target for OC patients. Additionally, CMRGPS can accurately predict the prognosis of OC patients and define the immunological milieu of OC patients, which can assist doctors in identifying specific patient subgroups who may benefit from immunotherapy and chemotherapy for specialized treatment.

## Data availability statement

Publicly available datasets were analyzed in this study. This data can be found here: https://www.jianguoyun.com/p/DRXvaEYQ0pH7ChiK6vMEIAA.

## Author contributions

SZ and JL conceived the study. SZ, XZ and FG drafted the manuscript. SZ performed the literature search and collected the data. JZ and HC analyzed and visualized the data. ZX, CC and JL helped with the final revision of this manuscript. All authors contributed to the article and approved the submitted version.

## Supplementary material

Supplementary Figure 1 Mutation analysis based on risk score models. **(A, B)** Waterfall plots summarizing mutations in patients in the high-risk and low-risk groups. **(C)** Differences in TMB between the high-risk and low-risk groups. **(D)** Kaplan-Meier curves for the four groups by risk score and TMB.

Supplementary Table 1 133 genes related to copper metabolism downloaded from MsigDB.

Supplementary Table 2 Univariate cox analysis and Kaplan-Meier survival analysis were used to select genes associated with copper metabolism that have prognostic significance for OC.

Supplementary Table 3 Queue information obtained for the two CM clusters.

Supplementary Table 4 40 prognostically relevant DEGs screened by univariate Cox analysis.

Supplementary Table 5 Detailed cohort information for the 2 CM gene clusters by the consensus clustering algorithm.
