# Supplementary figures and images for: Identification of copper metabolism-related subtypes and establishment of the prognostic model in ovarian cancer
Source: Front Endocrinol (Lausanne). 2023 Mar 6;14:1145797. doi: 10.3389/fendo.2023.1145797 (PMC10025496; doi:10.3389/fendo.2023.1145797)

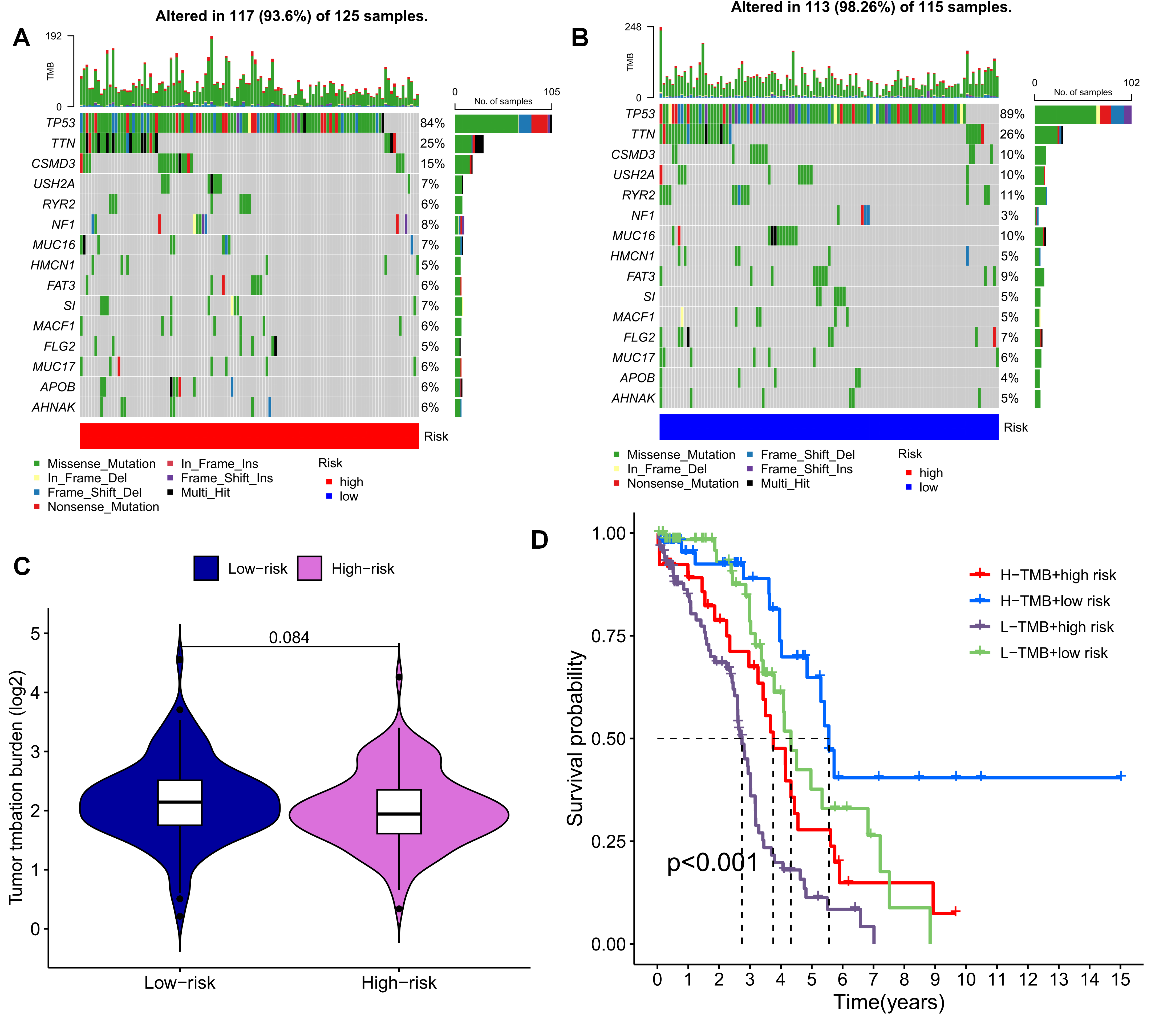

Supplement: Supplementary Figure 1 — Mutation analysis based on risk score models. (A, B) Waterfall plots summarizing mutations in patients in the high-risk and low-risk groups. (C) Differences in TMB between the high-risk and low-risk groups. (D) Kaplan-Meier curves for the four groups by risk score and TMB. [file Image_1.tif]
